# Presence of *Borrelia miyamotoi* infection in a highly endemic area of Lyme disease

**DOI:** 10.1186/s12941-020-00364-0

**Published:** 2020-05-30

**Authors:** Luis A. Marcos, Kalie Smith, Kelsey Reardon, Fredric Weinbaum, Eric D. Spitzer

**Affiliations:** 1 Division of Infectious Diseases, Department of Internal Medicine, Stony Brook University, 101 Nicolls Rd, HSC16-027 J, Stony Brook, NY 11794 USA; 2 Department of Microbiology and Immunology, Stony Brook University, Stony Brook, NY USA; 3 Stony Brook Southampton Hospital, Southampton, NY USA; 4 Department of Pathology, Stony Brook University, Stony Brook, NY USA

**Keywords:** *Borrelia miyamotoi*, Lyme disease, Tick borne diseases, New York

## Abstract

A series of cases in the Northeast of the US during 2013–2015 described a new Borrelia species, *Borrelia miyamotoi*, which is transmitted by the same tick species that transmits Lyme disease and causes a relapsing fever-like illness. The geographic expansion of *B. miyamotoi* in the US also extends to other Lyme endemic areas such as the Midwestern US. Co-infections with other tick borne diseases (TBD) may contribute to the severity of the disease. On Long Island, NY, 3–5% of ticks are infected by *B. miyamotoi*, but little is known about the frequency of *B. miyamotoi* infections in humans in this particular region. The aim of this study was to perform a chart review in all patients diagnosed with *B. miyamotoi* infection in Stony Brook Medicine (SBM) system to describe the clinical and epidemiological features of *B. miyamotoi* infection in Suffolk County, NY. In a 5 year time period (2013–2017), a total of 28 cases were positive for either IgG EIA (n = 19) or PCR (n = 9). All 9 PCR-positive cases (median age: 67; range: 22–90 years) had clinical findings suggestive of acute or relapsing infection. All these patients were thought to have a TBD, prompting the healthcare provider to order the TBD panel which includes a *B. miyamotoi* PCR test. In conclusion, *B. miyamotoi* infection should be considered in the differential diagnosis for flu-like syndromes during the summer after a deer tick bite and to prevent labeling a case with Lyme disease.

## Background

Suffolk County, a suburban county on Long Island (LI), New York, has a population of 1.8 million people and annually reports the highest absolute number of tick-borne diseases in NY. In 2017, there were 523 cases of Lyme disease, 55 cases of Ehrlichiosis, 31 cases of Anaplasmosis, and 138 cases of Babesiosis [1]. A series of cases in the Northeast US described a new Borrelia species, *Borrelia miyamotoi*, first reported to cause human infections in 2013 [2]. *B. miyamotoi* is closely related to the relapsing fever family of *Borrelia* spp (e.g. *B. hermsii*); however, it is transmitted by *Ixodes scapularis*, the same tick that transmits *Borrelia burgdorferi* (Lyme disease), *Anaplasma phagocytophilum* and *Babesia microti* [3]. The geographic expansion of *B. miyamotoi* in the US extends to other Lyme endemic areas such as the Midwest of North America [4]. *B. miyamotoi* infection can clinically present during warm months as a flu-like syndrome similar to Lyme disease, Anaplasmosis, or Babesiosis. The diagnosis of *B. miyamotoi* infection is complicated by the overlap in clinical manifestations caused by other TBD and the need to order specific diagnostic tests that may not be familiar to general practitioners even in hyperendemic areas for TBD such as LI [5]. In addition, little is known about co-infections between *B. miyamotoi* and the other TBD. Although 3–5% of ticks on LI have been found to be infected by *B. miyamotoi* and up to 74% have *B. burgdorferi* [6], there is no evidence that co-infections have occurred in humans who have been diagnosed with early Lyme disease in New York [7]. The goal of this study was to describe the recent epidemiology of *B. miyamotoi* by performing a retrospective chart review in all patients diagnosed with *B. miyamotoi* infection in Stony Brook Medicine (SBM) system, Suffolk County, NY.

## Methods

A retrospective study was conducted at Stony Brook Medicine (SBM) Hospitals (Stony Brook University Hospital–SBUH- and Southampton Hospital-SHH) between January 1, 2013 and December 31, 2017. SBM is the only tertiary medical center in Suffolk County, NY. The case search was performed from only positive tests results from the laboratory database. A positive result for *B. miyamotoi* was determined by either a positive real-time qPCR in the blood or IgG antibody detected by EIA using *B. miyamotoi* glycerophosphodiester phosphodiesterase recombinant antigen (rGlpQ) using previously described assays [8, 9] (performed at Oxford Immunotec, Norwood, MA).

## Results

At SHH, a total of 8575 PCR tests were performed for both *B. burgdorferi* and for *B. miyamotoi*, with positive results for 19 (0.2%) and 17 (0.19%) of these organisms, respectively. Other TBD results were; Babesia PCR in blood was 1.5% (263/17,626), *A. phagocytophilum* was 0.4% (80/17501), and *Ehrlichia chaffeensis* was 1% (172/16955). At SBUH, less than 200 PCR and IgG EIA tests for *B. miyamotoi* were performed during the study period. All PCR tests were negative at SBUH. For IgG EIA, 8 were positive at SHH (total tested = 38) and 11 were positive at SBUH (total tested = 60). A total of 28 cases were positive for either IgG EIA (n = 19) or PCR (n = 9) (8 other PCR-positive cases were not included because clinical information was not available).

All 9 PCR-positive cases (median age: 67; range: 22–90 years) had clinical findings suggestive of acute or relapsing infection. Of these 9 cases, 8 were men (88%), 3 were diagnosed in the outpatient clinic (33.3%), while the remaining 6 (66.6%) were diagnosed through the emergency room and required hospitalization. Demographics, clinical manifestations, and laboratory results on patients who had a positive blood *B. miyamotoi* PCR test are described in Table 1. None of these 9 cases had evidence for active co-infections with other TBD (all had negative blood PCR results for Ehrlichia, Anaplasma, Babesia, and *Borrelia burgdorferi*). One case had a positive IgM for Lyme with band 41 present in immunoblot but no erythema migrans, and another case had a positive IgG for *B. burdogferi* with 10 bands in the immunoblot.

**Table 1:** Demographics, clinical manifestations and laboratory results on patients with *Borrelia miyamotoi* PCR positive in the blood

Case	Age	Co-infections	Clinical manifestations	Laboratory findings					
	Gender			WBC (/mm^3^)	Hb (g/dL)	Platelets (/mm^3^)	Creatinine (mg/dL)	AST (IU/L)	ALT (IU/L)
1	90/M	Negative	Fatigue, vomiting, fevers	4100 (90% N)	9.7	91,000	1.46	74	46
2	22/M	Negative	Headaches, fevers, abdominal pain, arthralgia	3200 (88% N)	14.7	99,000	0.8	73	117
3	26/M	Negative	Fevers, diarrhea, hematuria	5400 (40%N, 30%B)	16.3	127,000	1.05	51	68
4	74/M	Negative	Fatigue, arthralgia	4600 (63% N)	14.2	154,000	0.7	21	28
5	32/M	Negative	Fevers, muscle pain, fatigue	3000 (45%N, 9%B)	15.6	166,000	1.0	98	65
6	74/M	Negative	Fevers, myalgia, chills, vomiting	6800 (N37%, B17%)	15.6	51,000	3.1	212	165
7	68/M	Negative	Fever, myalgia, arthralgia, fatigue	Unknown	Unknown	Unknown	Unknown	20	18
8	67/F	Negative	Fevers, arthralgias, mylagias	5500 (N 64%)	14.7	260,000	0.8	33	23
9	60/M	Unknown	Unknown Fevers, arthralgia, myalgias, fatigue	7100 (N 60%)	14.8	Unknown	Unknown	Unknown	Unknown

Case 1–6 were hospitalized. Case 7–9 were diagnosed in the outpatient clinic. All patients received doxycycline 100 mg orally for 14–21 days. Co-infections negative: Babesia, anaplasma and Ehrlichia PCR in blood were negative. Case 1: procalcitonin 0.19 ng/mL. Case 2: CSF analysis showed 14 WBC (86% L), Glucose 69 mg/dL, Protein 22 mg/dL Normal values: WBC (4800–10,800/mm3), Hb (12–16 g/dL), platelets (150,000-450,000/mm3), creatinine (less than 1.2 mg/dL), AST (less than 32 IU/L) and ALT (less than 33 IU/L)

*ARF* Acute renal failure, *CRP* C-reactive protein, *WBC* White blood cells, *N* Neutrophils, *B* bands, *M* monocytes, *L* Lymphocytes, *Hb* Hemoglobin, *AST* aspartate aminotransferase, *ALT* Alanine aminotransferase

### Discussion and conclusions

The positivity rate of *B. miyamotoi* PCR in this area of NY in a 5-year study period is 0.19% (17/8575). We were able to review clinical records for 9 of these PCR positive cases. *B. miyamotoi* PCR was ordered in these patients at SHH was because they had clinical manifestations compatible with a TBD in the summer and this PCR test is part of a TBD panel offered by a commercial laboratory. In contrast, at SBUH, diagnostic tests for *B. miyamotoi* (PCR and/or EIA) were rarely performed, most likely because the commercial TBD panel was not included in the routine test catalog; most of these tests were ordered as part of Infectious Disease service consultations, based on specific clinical findings.

It is possible that most of the patients in this study were thought to have Lyme disease after a deer tick bite, prompting the healthcare provider to order the TBD panel which includes a *B. miyamotoi* PCR test. One *B. miyamotoi* PCR positive case had a positive IgM for Lyme with the band 41 positive, which is not a serological criterion for acute Lyme. The presentation of this case was not consistent with acute Lyme, but with the other tick-borne disease such as Ehrlichia, Anaplasma or *B. miyamotoi* infection (leukopenia, transaminitis, thrombocytopenia). The true incidence of *B. miyamotoi* on LI is unknown; however, when compared to other TBD tested in the same healthcare system, the percentage of positive tests for *B. miyamotoi* was similar to that of other TBD present in this region. Lack of knowledge about laboratory tests for *B. miyamotoi* may hinder its detection. Whereas most clinicians are aware of the two-tiered algorithm for Lyme serology (despite its relatively poor sensitivity) [10], many clinicians are not aware that PCR performed on blood is the optimal test for detecting acute *B. miyamotoi* infection or that serologic diagnosis requires the use of rGlpQ-based EIA (the GlpQ protein is present in the relapsing fever group of Borrelia spp, but is not found in *B. burgdorferi*). *B. miyamotoi* infection often produces a positive result on a whole-cell lysate Lyme EIA or the Lyme C6 peptide antibody test [11, 12], but in most cases the Lyme immunoblot is negative, a result that is also common in early Lyme disease. Based on the partial cross-reactivity between *B. burgdorferi* and *B. miyamotoi*, a negative result on a Lyme serology assay does not rule out *B. miyamotoi* infection.

Limitations of this study include the retrospective nature and lack of convalescent titers in some patients. However, this study may serve first as a baseline to consider *B. miyamotoi* infection in some flu-like syndromes during the summer after a deer tick bite and second, to avoid misidentifying a case as early Lyme disease when in fact it could be a *B. miyamotoi* infection whose long-term symptoms may be similar to post-Lyme disease syndrome (PLDS) [13]. Whether some of the PLDS cases reported in clinical practice may have been related to *B. miyamotoi* infections or other TBD remains an open question.

## Data Availability

All data generated or analyzed during this study are included in this published article.

## Abbreviations

LI: Long island
PLDS: post-Lyme disease syndrome
SBM: Stony brook medicine
SBUH: Stony Brook University Hospital
SHH: Southampton Hospital
TBD: Tick borne disease(s)
